# Cholangiocyte Organoids in Liver Transplantation; a Comprehensive Review

**DOI:** 10.3389/ti.2024.12708

**Published:** 2024-07-19

**Authors:** C. Rejas, H. Junger

**Affiliations:** Department of Surgery, University Hospital Regensburg, Regensburg, Germany

**Keywords:** liver transplantation, cholangiopathy, cholangiocyte organoids, regenerative medicine, organoid implantation

## Abstract

Liver transplantation is the only curative option for many liver diseases that end up in liver failure, and cholangiopathy remains a challenging complication post-liver transplant, associated with significant morbidity and potential graft loss. The low availability of organs and high demand for transplantation motivate scientists to find novel interventions. Organoids, as three-dimensional cell cultures derived from adult cells or induced pluripotent cells, may help to address this problem. Different types of organoids have been described, from which cholangiocyte organoids offer a high level of versatility and plasticity for a deeper study of liver disease mechanisms. Cholangiocytes can be obtained from different segments of the biliary tree and have shown a remarkable capacity to adapt to new environments, presenting an effective system for studying cholangiopathies. Studies using cholangiocyte organoids show promising results for disease modeling, where organoids offer fundamental features to recapitulate the complexities of tissues *in vitro* and uncover fundamental pathological pathways to potentially reveal therapeutic strategies for personalized medicine. Organoids could hold the potential for regeneration of injured livers, representing tools of clinical impact in regenerative medicine when tissue damage is already present.

## Introduction

Liver transplantation (LT) remains the only curative treatment for end-stage liver disease (ESLD) [1]. Unfortunately, biliary complications occur in 5%–35% of the recipients after LT and remain elusive to curative strategies in this transplant procedure [1–4]. Indeed, post-LT cholangiopathies such as anastomotic (AS), non-anastomotic biliary strictures (NAS), and bile leakage [4, 5] represent the major causes of morbidity and graft failure after LT [4–7]. Efforts to avoid the occurrence of cholangiopathies have been researched, as it is the case with the implementation of Dual Hypothermic Oxygenated Machine Perfusion (DHOPE), which significantly reduced the impact of the cold storage in the liver by improving the preservation of the tissue [8]. However, severe cholangiopathies can lead to ESLD, with re-LT being the only curative option for these patients [6, 9].

Due to the shortage of donor livers for transplantation, novel therapeutic approaches to treat biliary diseases so as to prevent the need for transplantation and to treat biliary complications are crucial. Newly developed technologies and methods in regenerative medicine and stem cell research potentially offer novel treatment options, which can potentially rescue diseased bile ducts and thus make a liver transplant unnecessary. However, organoids can also be used to study and could potentially even be used to treat cholangiopathies that arise after LT.

Here, we present a comprehensive review focused on cholangiocyte derived organoids, including relevant insights into biliary anatomy, as well as the development, nomenclature, and molecular characteristics of cholangiocyte organoids. Furthermore, we provide the latest developments on the potential use of cholangiocyte organoids in disease modeling and treatment modalities.

## Biliary Anatomy and Post-Transplant Cholangiopathies

Derived from an endodermal bud, bile duct epithelium (BDE) is composed of cholangiocytes that line up to form an intricate network of branched conduits within the liver that constitute the biliary tree [10]. Closely associated with a branch of the portal vein and one or two branches of the hepatic artery, bile ducts are part of the classic view of a portal triad [11]. Stem (progenitor) cells are located intrahepatically in the canals of Hering, or extrahepatically in peribiliary glands (PBG) of the biliary tree [7]. These are called biliary tree stem cells (BTSCs), which are a set of multipotent cells that can differentiate into cholangiocytes, hepatocytes, or pancreatic islets [12].

During LT, severe damage to the biliary epithelium and PBG is associated with post-LT cholangiopathies [4, 13]. It has been shown that the PBG and its proliferating Sox9^+^ progenitor cells [14] drive bile duct regeneration. The BDE is highly vulnerable to ischemia, and although minor lesions often lead to re-epithelialization, more severe injuries can result in fibrosis and stricture formation [4, 15]. In general, injuries produced by ischemia (warm/cold ischemia, reperfusion injury, disturbed blood flow), immunological factors (ABO incompatibility, immune disease, virus infection, chronic rejection, chemokine mutations), or exposure to bile salts, can trigger post-transplant NAS cholangiopathies [16]. Damage to the PBGs and the vascular plexus strongly correlates with the development of NAS after transplantation, which is evident by poor regeneration of biliary epithelium due to progenitor cell destruction and insufficient blood supply [7]. Moreover, bile duct damage likely starts during the initial cold storage phase of the LT procedure [4]. When this damage progresses on a pathological level, poor persistent transplant function or loss is a common consequence, compared to transplanted organs that have no biliary damage [4, 14]. However, if a sufficient biliary regeneration of damaged bile ducts occurs in the early post-transplant phase, cholangiopathies can be prevented [17, 18].

## Cholangiocyte Organoids

To date, research on biliary injury is mostly based on two models: (i) rodent bile duct ligation, where an intrahepatic accumulation of bile leads to hepatotoxicity, cell proliferation, and fibrosis [19] to allow the study of injury repair mechanisms; and (ii) on histopathological examinations of human bile duct specimens, used to detect the molecular signatures of biliary insults [4, 5, 14]. However, both models have limitations. Animal models lack key components of human molecular mechanisms and are genetically homogenous, therefore these systems do not represent the specificity and diversity in humans [20]. The human specimen histological approach can be used to assess the morphological and molecular aspects of tissues, but this method captures only one moment in the disease course and is not amenable to dynamic therapeutic experiments [21].

The ability of cholangiocytes to adapt to new environments and the ease of culturing mature cells has recently brought bile duct organoids into focus. The concept of cultured organoids is constantly evolving, with the latest consensus defining organoids as *“self-organized, three-dimensional tissues that are typically derived from stem cells and embedded in a matrix, and which mimic the key functional, structural, and biological complexity of an organ”* [22]. However, as scientific knowledge rapidly expands, different variations of the term organoids arise, diversifying the already existing nomenclature. While the organoid concept is now well established, the types of organoids vary among research groups, complicating terminology overlap. In an important initiative to reduce this diversity, a consensus of terms was established to bring clarity and consistency in their use on organoids from hepatic, bile duct, and pancreas origin [23]. Three sub-classes of organoids have been defined: (i) epithelial organoids (lower complexity and higher reproducibility), (ii) multi-tissue organoids, and (iii) multi-organ organoids (higher complexity and lower reproducibility). Multi-tissue and multi-organ organoids do not harbor the capacity of self-renewal, but remain constant in a self-organizing structure; therefore, these models can be used to study, for example, cell-to-cell interactions and organ development. On the contrary, the simplicity and capacity for self-renewal of epithelial organoids, including cholangiocyte organoids, offer an excellent resource for studying bile duct biology and pathologies.

## Nomenclature

Cholangiocyte organoids derive from a variety of origins, depending on the starting biliary cell population. As previously described, these cells can be obtained from pluripotent stem cells [24] or differentiated cells [25]. Intrahepatic cholangiocyte organoids (ICOs) derive from the intrahepatic bile duct; studies have shown their ability to create branched structures, resembling smaller conduits of the biliary tree [26]. Extrahepatic cholangiocyte organoids (ECOs) derive from the common bile duct, gallbladder cholangiocyte organoids (GCOs) derive from the gallbladder [23], and finally, bile-derived organoids (BCOs) derive from extrahepatic cholangiocytes [27].

Diversity in gene expression among different biliary cell populations indicates functional variability depending on the biliary tree location, which aligns with the progression of cholangiocyte morphology through the bile ducts and their key role in bile secretion [11]. It is therefore evident that epigenetic regulations are involved, as cholangiocytes that are within or closer to the liver (as seen in intrahepatic and common bile duct organoids) are genetically more similar to each other than those derived from the gallbladder [25, 28–30]. Interestingly, these cells can modify their epigenetic imprinting to adapt to different stimuli [25]. This feature gives these cells transient and reversible characteristics that make their culturing useful for studying bile duct biology and for opening the way to potential cell therapy applications. For example, it has been shown that when subjected to specific culture methods, ICOs (but not ECOs) from human adult intrahepatic bile ducts can organize *in vitro* to form branching cholangiocyte organoids (BRCOs); BRCOs can maintain this architectural organization over several passages without displaying oncogenic activity. Further functional tests have confirmed the resemblance of BRCOs to those functions described for intrahepatic bile duct cells [26].

## Molecular Characteristics of Cholangiocytes and Organoids

Two distinct origins define cholangiocyte derivation. Intrahepatic cholangiocytes develop from bipotent progenitor cells called hepatoblasts, which initiate differentiation by expressing the transcription factors *SOX4* and *SOX9*, while extrahepatic cholangiocytes develop from the endoderm and express another set of transcription factors, including *PDX1*, *PROX1*, *HNF6*, *HNF1B*, *HEX1,* and *SOX17* [31]. Once differentiated, cholangiocytes express specific biliary markers such as *KRT7*, *KRT19*, *CFTR*, *HNF1B,* and *SOX9* [32]. Cholangiocytes within the liver also differ in gene expression profiles according to the close relation between their morphology and function: small cholangiocytes present a proliferative profile, while large cholangiocytes express genes associated with reabsorbing and secreting features [31]. At the same time, cholangiocytes have further differences in transcriptional patterns alongside the biliary tree, giving location specificity and functions over the chemical modification of bile [11, 28]. Despite these differences, it has been observed that small cholangiocytes can emerge from a rather dormant state to adapt to new stimuli, acquiring characteristics and functions of large cholangiocytes in response to hormones, chemical compounds, or mechanical stress such as that imposed by bile duct ligation [31]. Such plasticity has been studied in cholangiocyte organoids as well, where cultured cells stimulated to form organoids change their original genetic profile in response to specific stimuli. Moreover, these organoids can then resume functions when implanted in animal models of bile duct injury, even when placed in a region unrelated to their origin [28].

## Cholangiocyte Organoid Applications

As the understanding of organoid cultures advances, the diversity of research that this model can offer becomes larger [33, 34]. Organoids can act as mini-organs and allow the study of multiple aspects including developmental diseases, infectious diseases, genetic disorders, drug screening and testing, organ replacement therapy, cancer, organ transplantation, and regenerative medicine [35]. In this section, we will focus on organoids that are currently in use to study liver diseases.

Organoid culture methods have multiple advantages. Epithelial organoids are simple to handle, have great reproducibility, very low genetic variability, and enable scientists to quickly obtain data in living human cells [36]. In particular, the use of cholangiocyte organoids derived from adult bile ducts offers rapidly established cultures that begin to approach actual *in vivo* conditions that are desirable to recapitulate [33, 37]. Below, more details are discussed regarding various liver conditions that can be studied with organoid techniques.

### Ischemia-Reperfusion Injury (IRI)

Cholangiocytes are more sensitive than hepatocytes to hypoxia, particularly showing a significant decrease in viability after a re-oxygenation phase, potentially explaining the development of post-transplant cholangiopathies [38]. The impact of ischemia-reperfusion on the biliary tract is observed under the microscope as epithelial disruption, necrosis, decreased proliferation, fibrin deposition, and platelet aggregation [39]. In a recent study by Shi and colleagues, the exposure of intrahepatic cholangiocyte organoids (ICOs) to a hypoxic state for 72 h confirmed their hypersensitivity to low levels of oxygen by over-expressing the hypoxia-inducible factor 1-alpha (*HIF1a*) gene [40]. Cell-death pathway triggering is suggested by morphological changes including organoid size reduction, cytoplasmic disintegration, and nuclear fragmentation [40]. Interestingly, cells were able to recover after re-oxygenation, as shown by cell viability assays and confirmed by detection of the proliferation marker *Ki67*. Moreover, another study detected *VEGF* upregulation in small cholangiocytes, suggesting that angiogenic factors are involved in the ischemia response and that there might be close communication between the biliary epithelium and the surrounding endothelium [41]. Looking further into an insult stimulus, cholangiocytes enter a cell-death program mediated by tumor necrosis factor-alpha (*TNFα*), associated with necroptosis due to the inflammatory transcriptional profile exacerbated by the injury [42]. Cholangiocyte organoids are structures able to recapitulate biliary epithelium responses to injury, enabling a detailed study of pathways involved in liver lesions, including those induced by hypoxia [29, 40, 42, 43]. It is imperative to better understand the complex mechanisms underlying IRI, as this will lead to novel strategies to reduce damage caused through the liver donation process [40].

### Cholestatic Disorders

The mechanisms driving bile duct disruption and epithelial damage are still largely unknown, as these effects are the manifestation of complex and highly variable pathologies such as primary sclerosing cholangitis (PSC) and primary biliary cholangitis (PBC).

PSC is a heterogeneous disease characterized by biliary inflammation and progressive fibrosis of the intra and extrahepatic bile ducts [44]. Studies have shown that bile-derived organoids from PSC patients keep the disease features, as they continue to release specific cytokines *in vitro* [44]. PSC can also be associated with infections, for which organoids co-cultured with pathogens could provide an understanding of such interactions. A remarkable study on modeling the complexities of this disease was conducted by Zhang and colleagues, where multiple primary liver cells (cholangiocytes, hepatic stellate and liver endothelial cells) were established as multicellular organoids derived from PSC patients [45]. These PSC organoids showed key PSC gene alterations such as decreased expression of *EpCAM* and upregulation of *secretin*, as well as altered fibrosis markers (*ACTA2*, *COL1A1*, *desmin*, and *TGFB1*), angiogenic markers (*PECAM*, *CDH5*, and *von Willebrand factor*) and inflammatory markers (*IL6* and *TNFa*). Notably, these multicellular organoids were viable for up to 1 month and represent the first attempt to build organoids with different cell types, which will be critical to study cell-to-cell interactions in this disease [45].

PBC develops as a CD8^+^ T cell-mediated inflammatory bile duct injury, indicating a direct effect of these cells on the bile duct epithelium [46]. In a recent study, a co-culture organoid system was set up consisting of intrahepatic cholangiocytes and CD103^+^ T_RM_ cells (a subset of CD8^+^ T cells) derived from PBC patients [47]. Results suggested a direct cytotoxic effect on cholangiocytes evident from growth arrest and induction of cholangiocyte apoptosis [47]. Moreover, this detrimental effect of T cells on organoids was confirmed microscopically where damaged organoids showed an accumulation of T cells around them, which was also observed in 2D co-cultures and biopsies from PBC patients [47].

Alagille syndrome is a developmental disease of genetic origin that presents with mutations in the *JAGGED1* gene, disrupting the Notch-signaling pathway; it is characterized by apical polarity impairment in bile ducts leading to cholestasis [48]. Huch and colleagues established the first reported Alagille syndrome liver organoid system in 2015, where differentiated cells with a cholangiocyte profile derived from bi-potent liver cells showed an inability to integrate into the epithelium, detaching from the organoid into the lumen and becoming apoptotic [43]. Moreover, evidence shows that cholangiocyte organoids struggle with structural formation when the Notch-signaling pathway is blocked, which resembles the impairment that biliary cells undergo *in vivo* in Alagille syndrome [48, 49]. It has been proposed that organoids modeling the disease can be studied upon gene modifications with CRISPR-Cas9 methods to better understand or even correct the disease mutations [43], opening up novel paths for utilizing organoids in combination with other emerging technologies.

### Organoids in Regenerative Medicine

Regenerative medicine aims to restore, replace, or improve the functionality of injured or impaired tissues or organs as a therapeutic strategy applying a triad of concepts known as the R^3^ paradigm: replacement, regeneration and rejuvenation [50]. Efforts in this field started with the isolation of stem cells and their induction to pluripotency (induced pluripotent stem cells, iPSC) in hopes of finding personalized therapies using cells with a high potential to acquire features of different cell lineages [51]. However, high rates of mutations and DNA damage accumulations are well-known for iPSC in 2D-cell cultures, which raises concerns about the use of stem cells in cell therapy [52]. As the stem cell research field evolves, current demands surpass the 2D-cell culture limitations. Advancements will most likely require the inclusion of interactions of cell-to-cell and cell-to-matrix in a more realistic 3D level that helps to characterize pathologies spatially and dynamically [53]. To bring this knowledge to practical application in liver diseases, it is imperative to understand the liver’s natural regenerative mechanisms. For example, it was demonstrated by Raven and colleagues that impaired hepatocyte regeneration is necessary to trigger a ductal reaction that will allow cholangiocytes to adapt and differentiate into a hepatocyte-like phenotype as a way to compensate for cell injury [54]. This kind of tissue interaction can potentially be exploited in cell therapy based on organoids, as these complex cellular structures contain genetically stable cells, are highly functional, reproducible, and resilient to different microenvironments [55]. Another important aspect of regenerative medicine is the integration of gene editing in the organoid system to enable specific reprogramming and placement of dysfunctional cells in an autologous transplant situation [56]. However, while organoids are excellent tools for regeneration, they may not currently be suitable for patients in immediate need of treatment, since significant time is needed for their *ex vivo* development [28].

It has been shown that isolated human cholangiocytes that form organoids (ECOs) can be transplanted into the kidney capsule of NSG (immunodeficient) mice, demonstrating the ability of organoids to engraft and persist in animals for at least 12 weeks. Additionally, it has been shown that these ECOs are able to form tubular structures expressing *KRT19* [32]. These results prompted scientists to further explore the regenerative capacity of organoids, where gallbladder injury was induced in a mouse model; the injury was then surgically repaired by transplanting a biopolymer scaffold populated with ECOs. Later analyses showed full remodeling of the insult compared to controls that either died or formed fibrosis around the surgery [32]. Hallet and colleagues conducted a similar experience where human biliary epithelial cells (hBECs) were isolated from discarded human livers [57]. These cells were characterized and sorted by selecting CD133^+^ cells, a marker associated with hepatic progenitor cells [58]. Further characterization showed that organoids derived from these cells developed a transcriptional profile aligned with that of proliferating cholangiocytes (*Ki67* and *PCNA* staining). The hBECs were transplanted via intrasplenic injection into two immunocompromised mouse models that exhibit biliary disease or have IRI. The first model showed fewer intrahepatic lesions, decreased levels of bilirubin and fibrosis, and an increased animal survival rate versus controls. The IRI model also showed decreased bilirubin levels and reduced histological damage to the bile ducts with less necrosis than the untreated group. These results strongly suggest that hBECs help reduce the phenotype in these murine models and offer a potential regenerative response to biliary disease.

Post-transplant cholangiopathy is still a major clinical problem in LT [4, 6, 16] that might at least in part be induced by insufficient regeneration of damaged bile ducts [4]. A means to induce adequate regeneration and recovery of the biliary tree after LT remains evasive. However, with the emerging technology of liver *ex situ* normothermic perfusion (NMP), a novel model to study post-transplant cholangiopathies is applicable [59]. NMP provides adequate oxygen levels and temperature to the liver graft to ensure aerobic metabolism during the preservation stage [60]. This mechanism maintains cholangiocyte regeneration after reperfusion, as evidenced by a constant detection of the proliferation marker *Ki67* in intrahepatic bile ducts, therefore somewhat mitigating cellular insult to a mild injury [39]. In another remarkable experiment, Sampaziotis and colleagues demonstrated that cholangiocytes from different levels of the biliary tree can integrate into regions other than that of their origin and repair injured bile ducts [28]. Organoids derived from cholangiocytes of the gallbladder were injected into deceased (otherwise discarded) donor human livers with ischemic injury on the *ex situ* NMP for up to 100 h. The cells were delivered in a small area (a terminal branch of the intrahepatic ducts) to keep a high cell density. Organoids derived from cholangiocytes of the gallbladder engrafted and recovered up to 85% of the injured bile ducts. Upon analysis, these cells showed key biliary markers such as *KRT7*, *KRT19*, *CFTR*, and *GGT*, and lost *SOX17*, a gallbladder marker. After engraftment, these cells display an upregulation of intrahepatic cholangiocyte markers (*SOX4*, *BICC1*, and *DCDC2*), with no signs of differentiation into other hepatic cell types. At the end of the experiment, affected bile ducts presented both native and implanted cholangiocytes and no signs of cholangiopathy. After functional tests to assess the activity of the implanted cholangiocytes, it was determined that the bile composition was within normal parameters of volume and pH.

These outstanding findings reinforce the idea of using cholangiocytes as autologous transplants to repair the damaged biliary tract injury given their high plasticity, and support the idea of establishing biobanks with organoids readily available for allogeneic transplantation in case of liver transplantation and for patients with biliary diseases to prevent the need for transplantation in the first place.

## Discussion

LT has been for many years the only therapeutic alternative for patients presenting with ESLD. Burden on the healthcare system is immense and the aftercare of these patients is a challenge when cholangiopathies arise. An urgent need for new approaches to reverse or even avoid damage to the liver is ever-present.

The dynamic nature of cholangiocytes together with the development of organoid systems will be an important tool in a bright future for advances in hepatic pathology and liver transplantation research. Cholangiocyte organoids have shown great repairing capacity, opening a window for the detailed study and understanding of biliary tree pathologies, potentially preventing the development of ESLD and preventing transplantation. In an earlier stage of research, the application of organoids as a regenerative approach could aid in the treatment of several cholangiopathies that remain a major issue for patients with related complications after transplantation. The establishment of cholangiocyte organoids may be a valid option for the repopulation or regeneration of an insulted transplanted liver, using cultured cells derived from the same patient as autologous transplants, or even from donated cells in biobanks as allogenic transplants. Organoids can also be used to search for new therapeutic targets in drug screenings or find early therapeutic strategies to repair damage according to the mechanistic disease cause revealed through the utilization of these cultured structures. The three-dimensional structure of organoids allows cell-cell and cell-matrix interactive studies in a system that closely recapitulates physiological and physiopathological features of the liver. Since these cells can be induced to mimic specific diseases, their transcriptional and translational profiles will be useful to better characterize and understand pathological conditions such as IRI or cholestasis. The level of manipulation that organoids can tolerate is a key to studying the course of events in a given disease, providing specific information relevant to each patient, and allowing a better-informed strategy that could benefit individuals.

Taken together, these recent advances in cholangiocyte organoid culturing are likely to contribute to personalized medicine in LT. In addition, we have seen that the implementation of cholangiocyte organoids to alleviate complications affecting the biliary tree, brings to attention the possibility of decreasing liver transplant indications by preventing ESLD in the first place. This technology sheds light on a new approach for biliary tree pathophysiology, where the implementation of these cell cultures could rescue and help resume liver and biliary physiology in a more preventive fashion with minimal intervention.
